# Thymoquinone: A comprehensive review of its potential role as a monotherapy for metabolic syndrome

**DOI:** 10.22038/ijbms.2024.77203.16693

**Published:** 2024

**Authors:** Kasimu Ghandi Ibrahim, Shuaibu Abdullahi Hudu, Amina Yusuf Jega, Ahmad Taha, Abdurrahman Pharmacy Yusuf, Dawoud Usman, Kehinde Ahmad Adeshina, Zayyanu Usman Umar, Trevor Tapiwa Nyakudya, Kennedy Honey Erlwanger

**Affiliations:** 1 Department of Basic Medical and Dental Sciences, Faculty of Dentistry, Zarqa University, P.O. Box 2000, Zarqa 13110, Jordan; 2 School of Physiology, Faculty of Health Sciences, University of the Witwatersrand, 7 York Road, Parktown, 2193, Johannesburg, South Africa; 3 Department of Physiology, Faculty of Basic Medical Sciences, College of Health Sciences, Usmanu Danfodiyo University, Sokoto P.M.B 2346, Nigeria; 4 Department of Medical Microbiology and Immunology, Faculty of Basic Clinical Sciences, College of Health Sciences, Usmanu Danfodiyo; 5 University, Sokoto, Nigeria; 6 Department of Pharmaceutical and Medicinal Chemistry, Faculty of Pharmaceutical Sciences, Usmanu Danfodiyo University, P.M.B. 2254,; 7 Sokoto, Nigeria; 8 Department of Physiology, Faculty of Medicine, Port-said University, Egypt; 9 Department of Biochemistry, Federal University of Technology, P.M.B. 65, Minna, Niger State, Nigeria; 10 Biomedical Science Research and Training Centre (BioRTC), Yobe State University, Damaturu, Nigeria; 11 Department of Physiology, Faculty of Basic Medical Sciences, Federal University of Health Sciences, P.M.B. 45, Azare, Nigeria; 12 Department of Physiology, Faculty of Health Sciences, University of Pretoria, Private Bag X323, Gezina, 0031, South Africa

**Keywords:** Dyslipidemia, Metabolic syndrome, Nigella sativa, Obesity, Thymoquinone

## Abstract

Metabolic syndrome (MetS) is a widespread global epidemic that affects individuals across all age groups and presents a significant public health challenge. Comprising various cardio-metabolic risk factors, MetS contributes to morbidity and, when inadequately addressed, can lead to mortality. Current therapeutic approaches involve lifestyle changes and the prolonged use of pharmacological agents targeting the individual components of MetS, posing challenges related to cost, compliance with medications, and cumulative side effects. To overcome the challenges associated with these conventional treatments, herbal medicines and phytochemicals have been explored and proven to be holistic complements/alternatives in the management of MetS. Thymoquinone (TQ), a prominent bicyclic aromatic compound derived from *Nigella sativa* emerges as a promising candidate that has demonstrated beneficial effects in the treatment of the different components of MetS, with a good safety profile. For methodology, literature searches were conducted using PubMed and Google Scholar for relevant studies until December 2023. Using Boolean Operators, TQ and the individual components of MetS were queried against the databases. The retrieved articles were screened for eligibility. As a result, we provide a comprehensive overview of the anti-obesity, anti-dyslipidaemic, anti-hypertensive, and anti-diabetic effects of TQ including some underlying mechanisms of action such as modulating the expression of several metabolic target genes to promote metabolic health. The review advocates for a paradigm shift in MetS management, it contributes valuable insights into the multifaceted aspects of the application of TQ, fostering an understanding of its role in mitigating the global burden of MetS.

## Introduction

Metabolic syndrome (MetS) is a collection of linked metabolic risk factors that raise the predisposition to type 2 diabetes mellitus (T2DM), cardiovascular disease, and other non-communicable illnesses (1). As a global pandemic, MetS constitutes a significant financial and public health burden in both developing and developed countries (2). The prevalence of MetS has been on the rise, fuelled by factors such as sedentary lifestyles, poor dietary choices, and a rising prevalence of obesity. Typically, the occurrence of MetS tends to escalate with age, with a higher prevalence observed among individuals characterized by excess weight or obesity (3).

The prevalence of MetS and its associated cardio-metabolic components ranges from 12.5% to 31.4% globally and is notably greater in the Eastern Mediterranean Region and the Americas with a positive correlation with income levels (4). All metabolic disorders have seen an increase in prevalence rates, with high sociodemographic index (SDI) nations seeing the largest increases (5). Mortality rates linked with the components of MetS, specifically hyperlipidemia and hypertension, have declined over time, but not in T2DM and obesity (5). For the period spanning 2000 to 2019, the Eastern Mediterranean region of the World Health Organisation (WHO) and low to low-middle SDI countries had the greatest death rates from metabolic diseases (5). 

The current approach to treating MetS involves the targeting of the individual components of the syndrome separately (6). Hence a cocktail of drugs is used, e.g., statins for hyperlipidemia, angiotensin system inhibitors for hypertension, insulin-sensitizing agents for diabetes mellitus, etc. (7). However, this polypharmacy approach poses a significant challenge for several affected individuals due to the huge financial burdens and the potential for cumulative side effects associated with the prescribed medications. 

Natural products and herbal medicinal plants are believed to be safer than orthodox medications and are more readily available and affordable (8). WHO offers support and guidelines for the incorporation and use of natural products in the treatment of diseases (9). Many of these natural products have been fully characterized and their medicinal benefits documented. The efficacy of medicinal plant products in the management of various diseases has been attributed to the presence of their constituent bioactive phytochemical compounds (10). Among these phytochemicals is thymoquinone, an active ingredient from *Nigella sativa*. This review will comprehensively explore and discuss thymoquinone in the subsequent sections.

Thymoquinone (TQ) has been used traditionally in the treatment of many diseases, and several studies have also investigated and confirmed its benefits including beneficial effects against all the health outcomes associated with MetS (11). TQ has garnered significant attention from the scientific community for its potential therapeutic benefits such as its anti-oxidant properties (12), anti-inflammatory effect in mice (13), anti-cancer properties (14), immunomodulatory properties (15), neuroprotective effect in mice (13), cardio-protective effect in rats (16), and anti-metabolic syndrome in rats (hypoglycaemic, hypolipidaemic, and antioxidant actions) (17).

TQ with its broad spectrum of biological activities against the different components of MetS holds potential for use as a standalone treatment for MetS. This approach could eliminate the need for multiple pharmacological agents, thereby reducing associated costs and potential side effects. While the concept of a single-agent treatment for MetS is interesting, it entails careful consideration of some prospective challenges. Individuals with MetS exhibit varying combinations of different risk factors and varying degrees of severity (18). Moreover, a single-agent treatment might not be equally effective for everyone due to individual variations in genetic predisposition, lifestyle, and other factors. However, these risks are minimized or eliminated if the therapeutic agent is a natural product with multiple beneficial biological activities such as TQ. 

Several toxicological investigations show that TQ has a relatively broad safety margin. Acute and subacute toxicity evaluations in mice indicated a no observed adverse effect level (NOAEL) of about 10 mg/kg/day (19). Similarly, other studies have found no significant adverse effects in rodents following TQ administration, particularly in oral and nano-formulations (20). These reports collectively suggest that TQ has a relatively low toxicity profile, particularly when administered in appropriate doses and formulations.

In this article, we explore the potential beneficial activities of TQ against the different components of MetS, aiming to build a case for its consideration as a potential monotherapy for the condition. 

## Methods

Using Boolean operators, the authors systematically searched PubMed and Google Scholar for relevant studies. The search strings used include “thymoquinone AND metabolic syndrome”, “thymoquinone AND obesity”, “thymoquinone AND dyslipidemia”, “thymoquinone AND inflammation”, “thymoquinone AND insulin resistance”, “thymoquinone AND diabetes”, and “thymoquinone AND hypertension” and these terms were queried against the databases. The original research articles retrieved from the search were further screened for eligibility. Priority was given to articles published between 2016 and 2023 to provide authors with access to the most recent investigations on the effects of thymoquinone against MetS. After duplicate articles were removed, original research papers focusing on the effect of TQ on the individual components of MetS were included in this review.

**Thymoquinone: An overview**

***Introduction to thymoquinone and its natural sources***

**Figure 1:**
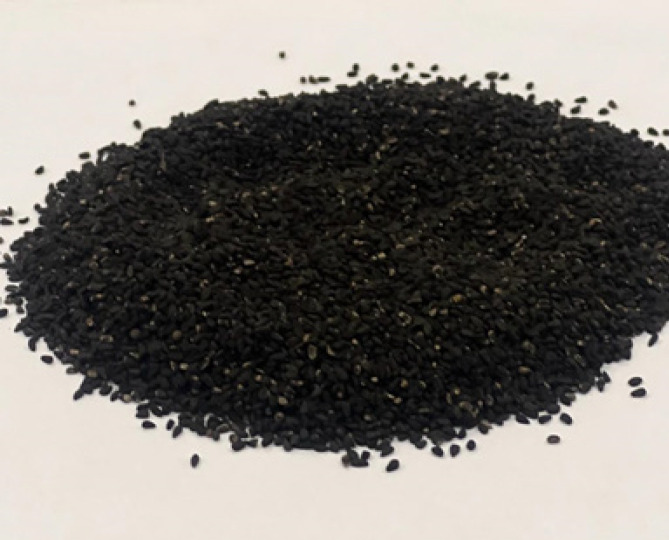
Picture of *Nigella sativa* (photo taken by authors 24/12/2023)

Thymoquinone (TQ) is a bioactive compound prominently found in various plant species, particularly those of the *Nigella* genus (21). It is abundantly present in the seeds of *N. sativa* L. (Figure 1), is popularly known as black cumin or black seed, and belongs to the family *Ranunculaceae* (22). *N. sativa* is an annual flowering plant native to Southwestern Asia, and its seeds have been used for centuries in culinary and traditional medicine practices in the Middle East, India, and other regions (23). These seeds are characterized by their distinctive dark color (24). The composition of *N. sativa* seeds includes a complex mixture of bioactive compounds, among which TQ stands out as a key component; its content in these seeds can range from 30 to 48 % of the seed’s volatile oil, highlighting its significance as a major constituent (25). 

***Chemical properties of thymoquinone***

**Figure 2:**
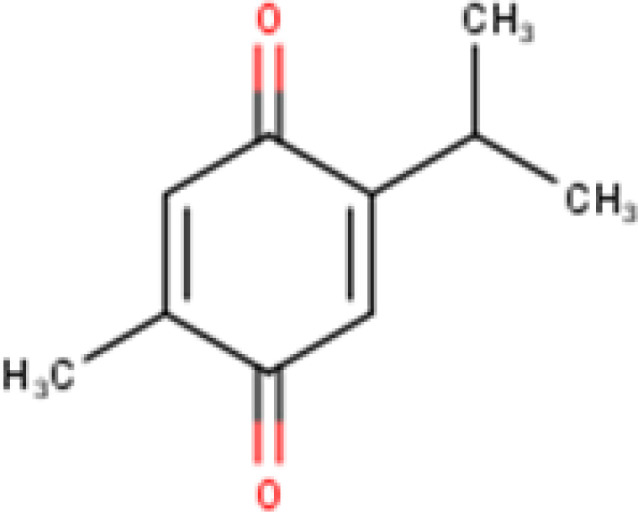
Chemical Structure of Thymoquinone (Source: Marvin Sketch by authors)

TQ is a naturally occurring compound with the chemical structure 2-isopropyl-5-methyl-1,4-benzoquinone (26). It is a bicyclic aromatic compound (Figure 2) that belongs to the class of quinones. The compound’s structure consists of a quinone ring system, characterized by two carbonyl groups, which confer its redox-active properties. TQ is known for its distinctive pungent taste and aromatic odor (27). 

***Pharmacokinetics of thymoquinone***

The chemical properties of TQ such as the quinone structure and its pharmacokinetic behavior including absorption, distribution, metabolism, and elimination collectively contribute to its bioactivity and potential health benefits. Thus, research previously conducted in that regard (28) summarises the following: when taken orally, TQ is absorbed through the gastrointestinal tract and distributed throughout the body via the bloodstream. However, TQ is insoluble in aqueous solutions, particularly at alkaline pH, which hinders its bioavailability (29). Its solubility is improved by mono-solvents such as Transcutol®, 2-butanol, and isopropanol and increases with temperature (30). Reports also show that nanoliposomes improve the solubility, stability, and bioavailability of TQ (31).

TQ has the potential to cross cell membranes due to its lipophilic nature, allowing it to interact with various tissues and cellular components. TQ undergoes metabolism in the liver. One of the primary metabolic pathways involves its reduction to dihydrothymoquinone, which is then further metabolized to various products, including conjugates with glutathione (32). These metabolites contribute to the compound’s overall bioactivity and potential health effects. Its metabolites are eliminated from the body mainly through urine and feces. Its elimination half-life can vary based on factors such as dose, route of administration, and individual variations in metabolism. For instance, Alkharfy *et al*. (28) reported an elimination half-life of 63.43±10.69 min for intravenous (IV) administration and 274.61±8.48 min for oral (PO) administration of TQ in rabbits.

***Traditional use of Nigella sativa seeds and modern applications of thymoquinone***

The primary natural source of TQ, *N. sativa* seeds, has a rich historical and cultural background, being employed for their medicinal properties in various traditional systems of medicine across different regions of the world. The seeds have been used for centuries in traditional medicine practices in the Middle East, Asia, and North Africa. In ancient Egypt, these seeds were discovered in the tomb of Tutankhamun, the ancient pharaoh, highlighting their importance to royalty at the time (33). Such historical backgrounds fostered their purported ability to address a wide range of health issues (34). 

Contemporary scientific research has explored and validated many of the historical uses of *N. sativa* seeds. Studies have identified TQ as the standout bioactive compound present in the seeds. The antioxidant, anti-inflammatory, antimicrobial, and anticancer properties of TQ have been investigated through various *in vitro* and *in vivo* studies (35). TQ, obtained from the seeds of *N. sativa *have been used to manage respiratory ailments such as asthma, bronchitis, and cough because they are believed to possess broncho-dilatory and anti-inflammatory properties that could help alleviate respiratory symptoms (35). The seeds are also used to address digestive discomfort, including indigestion, bloating, and gastrointestinal disturbances; they are thought to have carminative and antispasmodic effects that might help ease digestive issues (36). *N. sativa *seeds are used as immune system boosters and their potential immunomodulatory effect is believed to help the body combat infections and strengthen overall immunity (35). The anti-inflammatory property of TQ is utilized to manage inflammatory conditions, such as arthritis and joint pain because it is considered a natural remedy for pain relief (37). In terms of skin health, the seed has been used topically for skin conditions like eczema, psoriasis, and wound healing while the anti-inflammatory and antimicrobial attributes of TQ are thought to contribute to skin health (38). *N. sativa *seeds have been recognized for their potential antioxidant effects, which are believed to protect cells from oxidative stress and associated damage (37).

***Thymoquinone and obesity***

Obesity, one of the components of Mets (1), is a growing health problem of epidemic proportions, that is of serious concern because it also increases the predisposition of obese individuals to the development of other non-communicable diseases (NCDs), such as type 2 diabetes mellitus (T2DM), non-alcoholic fatty liver disease (NAFLD), kidney ailments, cardiovascular diseases such as stroke and cardiac failure (39) and some forms of cancer (40). Obesity is defined as a body mass index (BMI) greater than or equal to 30 kg/m^2 ^and results from an abnormal increase in the deposition of fat in adipocytes which then secrete adipokines that induce changes in the body (41). However, due to variations in body weight data across populations, obesity has conveniently been re-defined as a BMI in the 95^th^ percentile or more for a particular age group and sex based on the population data for that community (42). It is estimated that by the year 2030, about one billion people will be obese globally, with the prevalence being higher in lower- and middle-income countries compared to high-income countries (43). Thus, these alarming statistics underscore the importance of finding a natural treatment for obesity that will also work for the other components of MetS. 

**Figure 3:**
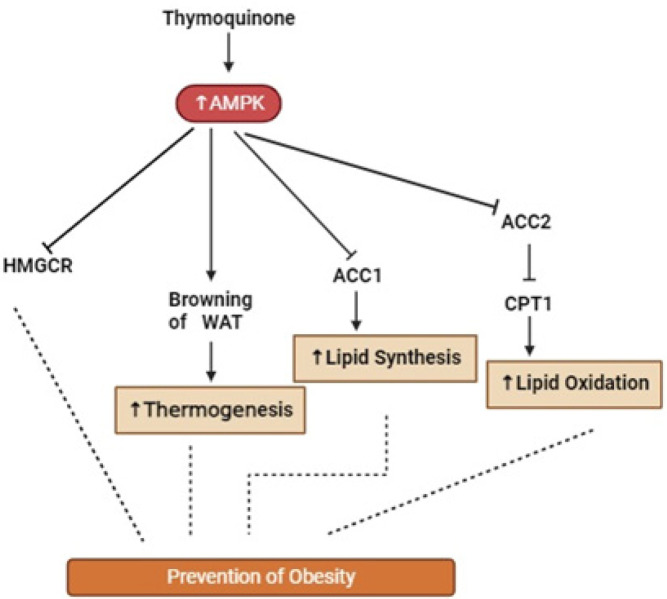
Anti-obesity effects of Thymoquinone

TQ has demonstrated potential for anti-obesity activity in both *in vitro* and *in vivo *studies. Like other natural polyphenols, TQ exerts its activity against metabolic disorders such as obesity through activation of 5’-adenosine monophosphate-activated protein kinase (*AMPK*) (44), which is key to the regulation of intracellular adenosine triphosphate concentration and hence, cellular metabolism (45). The *AMPK* pathway is essential in the treatment of obesity via its control of lipid metabolism through the regulation of two important lipid pathways, the carnitine acyl transferase (CPT)-1A and fatty acid synthase (*FAS*) pathways (46) (Figure 3).

TQ exhibited anti-obesity activity in an *in vitro *study of adipocyte differentiation. There was a decrease in lipid accumulation with increasing concentrations (6.25, 12.5, and 25 µg/ml) of TQ, caused by decreased expression of peroxisome proliferator-activated receptor gamma (*PPARγ*) (47). *PPARγ* regulates adipocyte differentiation through its downstream effect on the expression of *CCAAT*/enhance binding protein (*C/EBPα*) which then causes the subsequent activation of other genes involved in the process of adipocyte differentiation (48). Thus, TQ may exert its anti-obesity action through this *PPARγ/C/EBPα* pathway. 

In a Wistar rat model of olanzapine-induced metabolic abnormalities, TQ administered intraperitoneally at 10 mg/kg body weight alleviated the increased body weight, food intake, malondialdehyde levels, glutathione levels, and leptin-induced by olanzapine (49). TQ further increased the expression of AMPK proteins as determined using western blotting (49). 

Ghrelin is a polypeptide hormone discovered in 1999 that is synthesized in the stomach but exerts its activity in the brain, where it is involved in the regulation of appetite, body weight, and adiposity (50). Thus, increased ghrelin activity might directly lead to the development of obesity. When varying doses (1 mg, 2 mg, 10 mg, and 20 mg/kg) of TQ were administered to female Sprague-Dawley rats through the oral and intraperitoneal routes, it led to a decrease in the expression of ghrelin in the stomach of the rats (51).

In another study, when TQ (50 mg/kg, oral) was administered in combination with sage oil (*Salvia officinalis*) to high-fat-fed Wistar rats, it led to decreased weight gain and the final weight of the rats compared to the high-fat-only group (17), thus further demonstrating its potential to regulate weight in obese individuals. 

In a developmental programming study, streptozotocin-induced diabetic Swiss albino mice were administered TQ at 10 mg/kg daily during pregnancy and lactation to determine the effect of the intervention in their offspring. The pups from TQ-administered dams had decreased litter size and mean body weight (52), suggesting that TQ could program for decreased body weight in the offspring of diabetic mothers. This could be valuable for the management of both transgenerational diabetes and obesity if further investigated. 

A study (53) investigated the effect of administration of TQ on obesity in high-fat-fed male Wistar albino rats. Rats were fed a high-fat diet for 9 weeks and then treated with 10 mg/kg TQ daily for 6 weeks. TQ decreased the mean body weight and epididymal fat pad mass of the rats (53). 

The effect of TQ on the browning of white adipose tissue (WAT) was investigated both *in vitro* and *in vivo* (54). TQ reduced lipid droplet size and increased browning in 3T3-L1 cells and also decreased the level of inflammatory adipokines in the WAT of high fat-fed C57BL/6J mice (54). Browning of WAT refers to the transformation of white fat cells into cells that resemble brown adipose tissue (BAT), which is known for its thermogenic activity. This can increase energy expenditure and improve metabolic health. Several reports highlight the potential of WAT browning in improving metabolic disorders and protecting against obesity-related diseases (55). Because the process of browning of WAT is energy-demanding, it is considered important in mitigating obesity. The role of TQ in enhancing the browning of WAT may therefore be an important effect in the anti-obesity activity of TQ (Figure 3). 

Several other studies investigated the anti-obesity potential of *N. sativa* but not specifically thymoquinone, its most active component. We will present a few of them since the anti-obesity activity might be from the TQ in the preparations. 

In a study investigating the therapeutic potential of *N. sativa* against metabolic diseases including obesity, female mice were fed a high-fat diet and then administered varying doses of an aqueous extract of *N. sativa*. The extract improved obesity through decreased body weight, decreased fat formation, and adipocyte hypertrophy and also normalized fat metabolism through regulation of *AMPK* (56). *N. sativa* oil soft gelatin tablets at 450 mg were administered twice daily to 117 pre-diabetic human patients in a randomized study that compared *N. sativa* with Metformin (500 mg twice daily) and lifestyle modification. Participants who were administered *N. sativa* showed similar improvement in body weight, BMI, glycaemic control, improved lipid profile, and decreased expression of TNFα compared with the metformin group (57). The effect of *N. sativa* on systemic inflammatory markers in 90 volunteer obese women aged between 25 and 50 years was investigated in a double-blind, placebo-controlled randomized clinical trial. *N. sativa* decreased the serum concentration of TNFα and C-reactive proteins with no side effects reported (58). In another double-blinded, placebo-controlled trial investigating the efficacy of *N. sativa* on metabolic disturbances in central obese males, 1.5 g *N. sativa* powder administered for 3 months was found to decrease the body weight and waist circumference of the participants (59). 

Considering the above plethora of evidence (summarized in Table 1 below), TQ and *N. sativa* can be said to hold huge potential for development into a standardized anti-obesity agent that could also have efficacy against the other components of MetS. 

***Thymoquinone and dyslipidemia***

Dyslipidaemia, a metabolic disorder, is characterized by a dysregulated lipid profile, often presenting as elevated serum cholesterol, triglycerides, low-density lipoproteins (LDL), and very low-density lipoproteins (VLDL), coupled with a concomitant decrease in high-density lipoproteins (HDL) (60). Dyslipidaemia is one of the key metabolic defects that lead to MetS as it represents the major risk factor for insulin resistance, atherosclerosis, and cardiovascular diseases (60). Considering its strong lipid-lowering capacity, the role of TQ in managing dyslipidemia has recently gained attention (61). This section provides an overview of the modulatory effects of TQ on lipid profile, lipoprotein metabolism, and cholesterol synthesis and transport with emphasis on clinical evidence. 

***Effects of thymoquinone on lipid profile***

A study reported that the offspring of female mice that were diabetic during pregnancy and lactation had elevated blood lipids (HDL, LDL, and cholesterol) and increased risk of vascular complications of diabetes compared to the offspring of normal mothers (52). However, a 20 mg/kg/day oral supplementation with TQ in the diabetic pregnant and lactating dams significantly reduced the elevated lipid levels and mitigated the risk of developing diabetic complications in their offspring (52). In another study, a 100 mg/kg daily oral supplementation with TQ for one month in high-fat diet-fed mice resulted in a significant reduction in serum total cholesterol, triglycerides, LDL, and VLDL levels as well as an increase in HDL in the treated groups compared to non-treated control (62). Another study reported that a daily intraperitoneal injection with varying doses of TQ at 0.5, 1.0, and 2 mg/kg for 54 days resulted in a significant reduction in serum total cholesterol, triglycerides, and LDL levels in a rat model of bisphenol A-induced dyslipidemia (63). In a high-fat diet-treated mice model with a double knockout of the LDL receptor (LDL-R^−/−^), a significant elevation of serum lipids including TC, TG, and LDL was observed in the non-treated mice, and the serum levels of these lipids were significantly reduced by oral administration of TQ at 50 mg/kg/day (60). In another study, treatment with 25, 50, and 100 mg/kg oral TQ for 6 weeks prevented the elevation of TC and TG as well as a decrease in HDL in high-fructose diet-induced MetS in rats (64). 

Another study involving New Zealand white rabbits fed a high-cholesterol diet revealed that a 3.5 mg/kg daily oral supplementation with TQ significantly reduced the levels of serum TC, TG, and LDL and increased HDL in the treated rabbits compared to untreated controls fed the same diet (65). In another recent study, a 50 mg/kg/day oral supplementation with TQ alone or in combination with 0.052 ml/kg of sage essential oil resulted in a significant reduction in TC, TG, and LDL with a concomitant rise in HDL in HFD-treated rats compared to non-treated controls, with better outcomes in the combined treatment, indicating a possible synergistic effect of the combination (17). In streptozotocin (STZ)-induced diabetic rats, a 35 mg/kg/day oral supplementation with TQ for 5 weeks caused a significant decrease in TC, TG, and LDL as well as an increase in HDL in the treated groups compared to untreated diabetic controls (66). In a study involving a similar diabetic model, a 2 ml/kg oral supplementation with TQ-rich oil for 1 month led to a significant increase in serum HDL levels and a suppression of TC, TG, and LDL in the treated diabetic rats compared to untreated controls (67). In an HFD + STZ-induced type 2 diabetic (T2D) rat model, oral administration of TQ at 10 and 20 mg/kg daily for 2 weeks resulted in a significant decrease in serum TC, TG, and LDL and increased HDL in the treated groups compared to non-treated diabetic controls (68). In another STZ + nicotinamide–induced T2D rat model, a combined treatment with 10 mg/kg each of TQ and glycyrrhizin nano-formulations for 3 weeks acted synergistically to lower serum TG and VLDL levels in the treated diabetic rats compared to non-treated controls (69). This finding was mechanistically reaffirmed by *in silico* evidence which revealed that TQ could bind strongly to PPARγ, a key regulator of lipid metabolism (68). 

***Effects of thymoquinone on the metabolism and storage of cholesterol and lipoproteins***

In addition to improving lipid profiles, TQ could mitigate cardiovascular risk factors by modulating cholesterol and lipoprotein metabolism as well as their transport, uptake, and storage. In a study conducted to determine the effect of TQ on cholesterol synthesis and LDL uptake, human hepatic cell lines (HepG2 cells) were treated with either 2 µg/ml of commercially available TQ or 80 µg/ml of TQ-rich fraction from *N. sativa*. Both interventions resulted in a significant down-regulation of mRNA levels of HMGCR (a gene coding for HMG-CoA reductase, the rate-limiting enzyme in cholesterol biosynthesis) by 2- and 7-fold as well as a 71 and 12% up-regulation of LDLR (a gene coding for a family of proteins involved in the endocytosis of lipoproteins), respectively, in the treated cell lines compared to the untreated ones (70). Similarly, a twice-daily supplementation with 0.5 ml of 10 mg TQ for 30 days ameliorated cardiovascular risk factors by modulating cholesterol and lipoprotein metabolism in rats, fed an atherogenic suspension of LDL (71). According to the authors, TQ treatment inhibited HMG-CoA reductase and increased the activity of arylesterase (an inhibitor of oxidative damage in lipoproteins that correlates positively with HDL levels)(71, 72). The treatment also prevented the shift in the buoyancy of LDL from the less atherogenic large buoyant-LDL (lb-LDL) to the more atherogenic small dense-LDL (sd-LDL) and restored the normal distribution of LDL and apoB into lb-LDL and sd-LDL to nearly normal levels (71). In another study involving the same rat model, oral supplementation with 100 mg/kg TQ-rich methanolic extract or 20 mg/kg volatile oil of *N. sativa* for 30 days resulted in a significant reduction in TC, TG, VLDL, and LDL and its subscriptions (lb-LDL and sd-LDL) as well as an increase in HDL in the treated rats compared to non-treated hyperlipidaemic controls. This was accompanied by a decreased HMG-CoA reductase and increased arylesterase activities leading to reduced cholesterol synthesis and lipid peroxidation (73). A study involving apolipoprotein E knockout (ApoE^-/-^) mice fed a high-cholesterol diet and supplemented orally with 25 mg/kg/day of TQ FOR 8 weeks reported a significant down-regulation of the lectin-like oxidized low-density lipoprotein receptor-1 (LOX-1) mRNA and protein expression as well as significantly reduced serum levels of TC, TG, and LDL (74). LOX-1 is a scavenger receptor that is involved in the initiation of atherosclerosis due to its role in the uptake of oxidized LDL into endothelial cells which leads to endothelial plaque formation (75). The animal studies have shown clinical translatability in humans as will be discussed further.

***Clinical evidence supporting thymoquinone’s impact on dyslipidemia***

A recent randomized, double-blind, placebo-controlled, clinical trial evaluated the benefits of TQ-rich *N. sativa* seed oil in reducing cardiovascular risks in hypertensive patients. The study reported that twice daily supplementation with 2.5 ml of the pure TQ-rich oil for eight weeks resulted in a significant reduction in serum total cholesterol and LDL levels and an increase in HDL in the treated subjects compared to a placebo group receiving the same amount of sunflower oil (76). A more recent phase I clinical trial involving healthy volunteers to evaluate the safety of TQ-rich *N. sativa* seed oil (5.2% v/v TQ content) reported that treatment with 200 mg/adult/day TQ orally for 3 months caused a significant reduction in serum TC, TG, LDL, and VLDL levels in the treated subjects compared to the untreated volunteers (77). In non-alcoholic fatty liver disease patients, daily oral supplementation with 1 g TQ-rich oil for two months resulted in a significant reduction in total cholesterol, triglycerides, LDL, and VLDL as well as an increase in HDL levels in the treated group compared to a placebo receiving an equal amount of paraffin oil (78). Table 2 summarizes the research findings on the role of TQ in modulating dyslipidemia and the risk of MetS.

***Thymoquinone and hypertension***

Hypertension arbitrarily refers to sustained elevated blood pressure above 140/90 mmHg in an individual with the measurements taken at least at two or more different contact times (79). Hypertension is an important component of MetS (80), while MetS itself is a predisposing factor for other cardiovascular diseases such as myocardial infarction, atherosclerosis, and stroke (1). Other components of MetS such as obesity and IR also contribute significantly to the development of hypertension (81), further worsening the situation. Uncontrolled hypertension leads to end-organ damage, especially in the kidneys, eyes, heart, and brain (82). 

**Figure 4:**
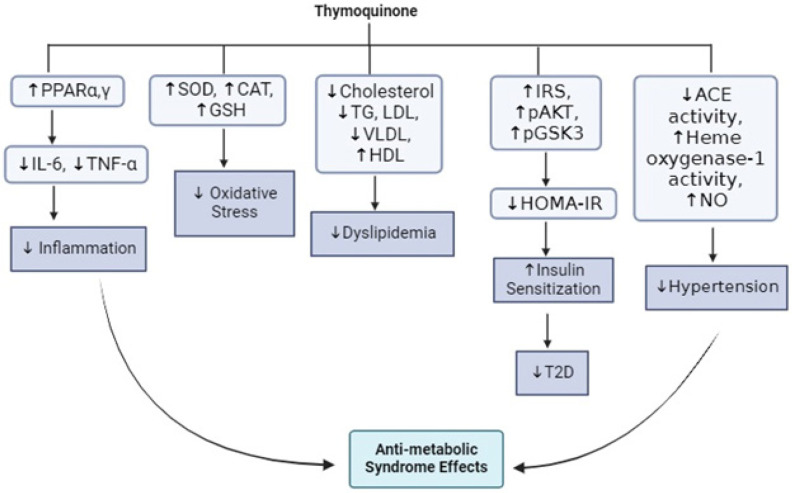
Effects of thymoquinone on the individual components of metabolic syndrome

TQ directly or indirectly as a constituent of *N. sativa* seeds (extracts) has shown a lot of promise in the treatment of hypertension as reported in several studies shown in Table 3 below. Some of the possible mechanisms of action proposed include antioxidant, decreased cardiac oxidative stress (17), angiotensin II receptor blockage (83), decreased angiotensin-converting enzyme activity (84), vascular muscarinic activity (85), central acting, calcium channel blockage, and diuretic activity (86)(Figure 4).

***Thymoquinone and insulin resistance***

Insulin resistance (IR), one of the key components of MetS is a metabolic condition wherein peripheral tissues such as the liver, adipose tissue, and skeletal muscles fail to respond adequately to normal insulin levels in the bloodstream, despite sufficient insulin secretion by pancreatic β-cells (99). This reduced responsiveness often stems from defects in the insulin signaling pathway, commonly induced by oxidative damage to key proteins involved in the signaling cascade and or inflammatory responses that alter their expression and function (100). Persistent IR can lead to β-cell exhaustion, leading to hypoinsulinemia and ultimately T2DM (101). Biochemical manifestations of IR encompass hyperinsulinemia, hyperglycemia, glucose intolerance, dyslipidemia, and dysregulated levels of adipokines in the blood (102). Preliminary biochemical tests to assess IR include homeostasis model assessment of insulin resistance (HOMA-IR), oral glucose tolerance test (OGTT), and insulin tolerance test (ITT)(103). 

Due to its outstanding anti-oxidant and anti-inflammatory properties, the potential of TQ in the treatment of IR is emerging, particularly in mitigating the unfavorable alterations in insulin signaling caused by oxidative stress and inflammation (104). This section provides a brief overview of the role of TQ in modulating insulin signaling, glucose metabolism, adipokines, and inflammation as well as the clinical implications of interventions with TQ in IR and T2DM. 

***Influence of thymoquinone on insulin signaling pathways and glucose metabolism***

Studies have reported improved insulin sensitivity, decreased IR, and enhanced glucose metabolism in murine models of MetS and T2D (105). In one of these studies, oral supplementation with 25, 50, and 100 mg/kg of TQ increased insulin sensitivity and glucose tolerance by enhancing the expression of PPAR-α and PPAR-γ in a rat model of high-fructose diet-induced MS (64). PPAR-γ is reported to improve insulin sensitivity and glucose uptake in adipocytes by up-regulating GLUT-4 expression and increasing its translocation to the cell membrane surface (106). Moreover, PPAR proteins particularly PPAR-α and PPAR-γ are known to play important roles in lipid breakdown and fatty acid oxidation and hence protect against free fatty acid-induced inflammation and IR (107). In a study involving mice with diet-induced obesity, treatment with 20 mg/kg TQ orally for 24 weeks resulted in a significant improvement in insulin signaling and glucose tolerance as evidenced by enhanced OGTT and ITT as well as increased protein expression of phosphorylated Akt (pAkt, a key component of the insulin signaling pathway) via SIRT-1/AMPKα-dependent signaling (108). In another study, dietary supplementation with 400 µl/kg TQ for 7 months prevented IR associated with the chronic use of highly active antiretroviral therapy drugs used to treat HIV-1 infection such as nelfinavir, zidovudine, and efavirenz (109). In STZ-induced diabetic rats, it was reported that oral supplementation with 50 mg/kg TQ for 1 month resulted in up-regulated protein expression and phosphorylation of pAkt in the cardiac muscle of the treated rats compared to non-treated controls (110). In HFD-fed mice, dietary supplementation with 0.75% TQ in combination with 2% Omega 3 (ω3) fatty acid resulted in significantly up-regulated protein expression of the hallmarks of white adipose tissue (WAT) browning such as UCP1, PRDM16, FGF21, and the phosphorylated forms of the key components of the insulin signaling pathway (pAkt and pIRS1) in the adipose tissue of the treated mice compared to untreated controls. The authors also reported increased phosphorylated pIR, pIRβ, and pIRS1 in the liver of the treated mice (54). In a rat model of MetS fed a western diet high in fat and cholesterol and treated with 10 and 20 mg/kg of TQ, a significant reduction in IR was observed as evidenced by decreased homeostasis model assessment of insulin resistance (HOMA-IR) (111). Significant reductions in HOMA-IR values were also reported in a rat model of HFD-induced MetS treated orally with a combination of 50 mg/kg TQ and 0.052 ml/kg sage essential oil for 10 weeks (17) and an HFD and STZ-induced T2D rats treated with 10 and 20 mg/kg daily oral TQ for 2 weeks (68). In rats bisphenol A (BPA)-induced MS treated with 0.5, 1, and 2 mg/kg of TQ or 21, 42, and 84 μl/kg of TQ-rich *N. sativa* oil intraperitoneally for 54 days, the treatment led to up-regulated protein expression of pIRS, pAKT, and pGS3K in the treated rats compared to non-treated controls (63). We may imply from these findings that TQ could enhance insulin signaling and glucose metabolism by ameliorating the MetS-induced perturbations in the insulin signaling pathway and glucose transport (Figure 4). Hence, the bioactive compound could be useful in managing T2D and related sequelae associated with MetS.

***Impact of thymoquinone on adipokines and inflammation***

Studies have provided valuable insights into the ameliorative benefits of TQ against inflammation as a key component of MetS mediated by adipocyte secretions in obese and non-obese models of MetS. In one of the studies, daily oral supplementation with 20 mg/kg/ TQ for 24 weeks in a mouse model of diet-induced obesity resulted in significantly reduced serum levels of inflammatory adipokines (resistin and MCP-1) in the treated mice compared to non-treated controls (108). In HFD-fed mice, a significant reduction in the inflammatory adipokine NOV/CCN3 was observed in the adipose tissue and liver of HFD mice supplemented with a combination of 0.75% and 2% ω3 fatty acid in the diet (54). In a rat model of olanzapine-induced MS, treatment with 2.5, 5, or 10 mg/kg TQ ameliorated the elevation of serum leptin levels caused by olanzapine administration (49). 

Serum elevation of leptin (particularly in obese conditions) is associated with inflammation and IR (112). In BPA-induced MetS, a significant decrease in the protein content of leptin, IL-6, and TNF-α was observed in the liver of rats injected intraperitoneally with 0.5, 1, and 2 mg/kg of TQ or 21, 42, and 84 μl/kg of TQ-rich *N. sativa* oil for 54 days compared to rats exposed to BPA without treatment. In the human THP-1 cell lines, treatment with 5 and 10 µM of TQ reduced the risk of atherosclerosis by mitigating inflammatory responses via down-regulation of the protein expression of MCP-1 and ICAM-1 in response to IFN-γ in the treated cell lines (113). It could be inferred from these observations that TQ may ameliorate IR, cardiovascular risks, and other components of MetS by mitigating the serum and tissue levels of inflammatory adipokines. Hence, TQ could be a promising therapeutic alternative for patients with MetS.

***Clinical implications for insulin resistance and type 2 diabetes***

Currently, there is a dearth of clinical data on the ameliorative benefits of TQ against IR and T2D. However, a recent systematic review of clinical studies has reported the ameliorative benefit of various TQ-rich preparations of *N. sativa* seeds against IR in patients with T2D and related sequelae (114). Table 4 summarizes the role of TQ in insulin signaling, glucose metabolism, and the serum and tissue levels of inflammatory adipokines.

***Safety profile of thymoquinone***

TQ has gained attention for its potential health benefits, but as with any natural compound, it is essential to assess its safety profile to ensure its appropriate use. Research has been conducted to evaluate the safety of TQ, both in traditional medicine practices and modern scientific investigations (115). The historical use of *N. sativa* seeds in various cultures indicates a lack of significant reports of adverse effects (20, 115). Nevertheless, it is important to note that traditional use does not guarantee safety, and individual responses may vary. To ascertain potential harmful effects, acute and sub-chronic toxicity studies involving animals have been conducted, administering varying doses of thymoquinone and monitoring for adverse effects on various organs and physiological parameters. Reports indicate the LD50 values of 250–794 mg/kg in rats and 300–2400 mg/kg in mice for oral TQ; 57 mg/kg in rats; and 90.3–104 mg/kg in mice for intraperitoneal TQ (20). More so, NOAEL for TQ is about 10 mg/kg (19, 20). These findings suggest a relatively low toxicity profile at therapeutic doses and have supported the use of TQ in clinical trials (77). TQ has also been evaluated for its potential to cause genetic mutations (mutagenicity) or damage to DNA (genotoxicity). The results of these studies have generally been negative or inconclusive, suggesting that thymoquinone is unlikely to induce significant genetic damage (116).

While TQ offers potential health benefits, it’s essential to be aware of potential adverse effects and take necessary precautions when considering its use (117). Some potential adverse effects and precautions associated with thymoquinone include gastrointestinal distress, allergic reactions, and interactions with medications (118). 

***Future directions***

In the exploration of TQ as a potential therapeutic agent for MetS, it is crucial to acknowledge the current gaps in our understanding. While substantial research has revealed its positive effects, there are still several areas that need further investigation. Firstly, the long-term safety profile of TQ, especially at higher doses, warrants comprehensive evaluation and assessment to ensure its safety in clinical settings. Moreover, the optimal dosage and duration of thymoquinone supplementation for various aspects of MetS require clarification. Additional research should also concentrate on identifying specific patient populations with metabolic dysfunction that might gain the most from thymoquinone treatment. Mechanistic research studies into the precise pathways through which thymoquinone exerts its effects need to be investigated to facilitate the development of targeted therapies and medicines.

The possibility of combination therapy and synergistic effects with thymoquinone emerges as a promising route in the search for treatments for MetS. Future studies should examine how thymoquinone interacts with other pharmaceuticals, dietary supplements, or lifestyles in addressing the multifaceted nature of MetS. Investigating the compatibility of thymoquinone with currently available medications commonly prescribed for metabolic syndromes and its risk factors, such as statins, anti-hypertensive drugs, or anti-diabetic pharmacological agents, may reveal innovative and novel treatment approaches that maximize therapeutic outcomes while minimizing side-effects.

Throughout this review, we have highlighted and emphasized on the mounting evidence supporting the potential of thymoquinone in mitigating various aspects of MetS. The diverse effects of thymoquinone on obesity, dyslipidemia, hypertension, and insulin resistance highlight its potential as a putative therapeutic agent for this challenging condition. Its anti-oxidant and anti-inflammatory qualities, combined with its ability to modify cellular signaling pathways, provide a strategy for tackling the underlying causes of MetS. However, it is crucial to understand that translating these promising findings into clinical practice will require rigorous clinical trials and continued research.

Activation of AMPK by thymoquinone exerts antiobesity effects by inhibiting ACC1, which is responsible for fatty acid synthesis. AMPK also enhances fatty acid oxidation by counteracting the inhibition of CPT1 (carnitine palmitoyltransferase 1) caused by malonyl-CoA. In addition, thymoquinone stimulates WAT browning which enhances thermogenesis thereby increasing energy expenditure. Lastly, AMPK phosphorylates and inhibits HMGCR, which along with its effects on ACC1 and ACC2, causes a preprogramming of lipid and sterol synthesis in the cell

ACC1- Acetyl CoA Carboxylase 1; ACC2- Acetyl CoA Carboxylase 2; AMPK- AMP-activated protein kinase; CPT1- Carnitine palmitoyltransferase 1; HMGCR- 3-hydroxy-3-methyl-glutaryl-coA reductase; WAT- White adipose tissue

ACE: Angiotensin converting enzyme; CAT: Catalase; GSH: Reduced glutathione; HDL: High-density lipoproteins; HOMA-IR: Homeostasis model assessment of insulin resistance; IL-6: Interleukin 6; IRS: Insulin receptor substrate; LDL: Low-density lipoproteins; NO: Nitric oxide; pAKT- Phosphorylated protein kinase B; pGSK3: Phosphorylated glycogen synthase kinase-3; PPARα: Peroxisome proliferator-activated receptor alpha; PPARϒ: Peroxisome proliferator-activated receptor gamma; SOD: Superoxide dismutase; T2D: Type 2 diabetes; TG: Triglyceride; TNF-α: Tumour necrosis factor alpha; VLDL: Very low-density lipoproteins

**Table 1:** Summary of studies reporting on the anti-obesity activity of thymoquinone

Experimental model	Dose and duration	Findings	Reference
*In vitro* Evidence			
Human adipose tissue-derived Stem Cells (ADSCs)	TQ: 6.25, 12.5, and 25 µg/ml for 21days in cell culture	Decreased lipid accumulation, reduced FAS/β-actin ratio, decreased expression of PPARγ	(47)
3T3-L1 cells	TQ: 1-4µM for 6 days	Reduced droplet size	(54)
*In vivo* Evidence			
Olanzapine-induced metabolic dysfunction in Wistar rats	TQ: 10 mg/kg/day intraperitoneal for 15 days	Decreased body weight and feed intake decreased AMPK proteins	(49)
Female Sprague Dawley rats	TQ: 1 mg, 2 mg, 10 mg/kg intraperitoneal, and 20 mg/kg orally for 42 days	Decreased expression of ghrelin in the stomach	(51)
High-fat-fed Wistar rats	TQ: 50 mg/kg, orally for 10 weeks	Decreased weight gain and final body weight	(17)
Streptozotocin-induced diabetic Swiss albino mice	TQ: 20 mg/kg/day orally throughout pregnancy and lactation	Decreased litter size and body weight Significantly restored levels of blood glucose, insulin, free radicals, plasma cytokines, and lipids as well as lymphocyte proliferation in the offspring	(52)
High fat-fed male Wistar albino rats	TQ: 10 m/kg/day intraperitoneal for 6 weeks	Decreased mean body weight Decreased epididymal fat pad	(53)
High fat-fed C57BL/6J mice	0.75 % TQ in dietary combination with 2% ω3 fatty acid for 8 weeks	Up-regulated protein expression of adipose tissue browning markers and insulin signaling components	(54)
45% Kcal-fed obese mouse model	Black cumin seed extract: 400, 200, and 100 mg/kg orally for 84 days	Decreased body weight Dose-dependent decreased abdominal and body fat accumulations, decreased adipocyte hypertrophy	(56)
Clinical Trial			
Obese pre-diabetics	*N. sativa* oil soft gelatin capsules 450 mg twice daily orally	Decreased body weight, BMI, lipid profile, inflammatory markers, TNF-α, SIRT 1, and P53	(57)
Obese women	Low-calorie diet with 3 g/day of NS oil for 8 weeks	Decreased TNF-α and C-reactive proteins	(58)
Obese males	1.5 g *N. sativa* powder orally for 3 months	Decreased body weight and waist circumference	(59)

TQ: Thymoquinone, FAS: fatty acid synthase, PPAR: peroxisome-proliferator activated receptor γ: gamma, α: alpha, AMPK: adenosine monophosphate protein kinase, TNF: tumor necrosis factor, SIRT 1: sirtuin 1, p53: tumor protein 53

**Table 2:** Role of thymoquinone in modulating dyslipidemia, cholesterol, and lipoprotein metabolism

Study model	Treatment and dosage	Treatment outcomes	References
Effects on Lipid Profile			
Diabetic pregnant and lactating mice	20 mg/kg/day oral TQ during gestation and lactation	Reduced elevated blood lipids (HDL, LDL, and cholesterol) and lowered the risk of diabetic complications in offspring	(52)
High-fat diet-treated mice	100 mg/kg daily oral TQ for 1 month	Significant reduction in serum total cholesterol, triglycerides, LDL, and VLDL, with an increase in HDL	(62)
Rat model of bisphenol A-induced dyslipidemia	Intraperitoneal injection of TQ at 0.5, 1, and 2 mg/kg for 54 days	Significant reduction in serum total cholesterol, triglycerides, and LDL levels	(63)
High-fat diet-treated LDL receptor knockout mice (LDL-R−/−)	50 mg/kg/day oral TQ for 8 weeks	Significant reduction in serum total cholesterol, triglycerides, and LDL levels	(60)
High-fructose diet-induced metabolic syndrome in rats	25, 50, and 100 mg/kg oral TQ for 6 weeks	Prevention of elevated total cholesterol and triglycerides, along with maintaining HDL levels	(64)
New Zealand white rabbits fed a high-cholesterol diet	3.5 mg/kg daily oral TQ	Significant reduction in serum total cholesterol, triglycerides, and LDL, along with increased HDL levels	(65)
High-fat diet-treated rats	50 mg/kg/day oral TQ alone or in combination with 0.052 ml/kg of sage essential oil for 10 weeks	Significant reduction in total cholesterol, triglycerides, and LDL, and an increase in HDL levels with better outcomes in the combined treatment	(17)
STZ-induced diabetic rats	35 mg/kg/day oral TQ for 5 weeks	Significant decrease in total cholesterol, triglycerides, and LDL, and an increase in HDL levels	(66)
STZ-induced diabetic rats	2 ml/kg oral TQ-rich oil for 1 month	Increased HDL levels and suppression of total cholesterol, triglycerides, and LDL	(67)
STZ + high-fat diet-induced type 2 diabetic rats	10 and 20 mg/kg daily oral TQ for 2 weeks	Significant decrease in total cholesterol, triglycerides, and LDL, along with increased HDL levels	(68)
STZ + nicotinamide-induced type 2 diabetic rats	Combined treatment with 10 mg/kg each of TQ and glycyrrhizin nanoformulations for 3 weeks	Lowering of serum TG and VLDL levels	(69)
Effects on Cholesterol and Lipoprotein Metabolism			
*In vitro* Evidence			
Human hepatic cell lines (HepG2 cells)	2 µg/ml of TQ or 80 µg/ml of TQ-rich fraction from *N. sativa*	Down-regulation of *HMGCR* mRNA and up-regulation of *LDLR* mRNA in the treated cell lines	(70)
*In vivo* Evidence			
Rats fed an atherogenic suspension	Twice-daily supplementation with 0.5 ml of 10 mg oral TQ for 30 days	Inhibition of HMG-CoA reductase, increased arylesterase activity, prevention of LDL shift from lb-LDL to sd-LDL, and restoration of normal LDL distribution	(71)
Rats fed an atherogenic suspension	100 mg/kg oral TQ-rich methanolic extract or 20 mg/kg volatile oil of *N. sativa* for 30 days before atherogenic suspension	Reduction in TC, TG, VLDL, and LDL, increase in HDL, decreased HMG-CoA reductase activity, and increased arylesterase activity	(73)
Apolipoprotein E knockout (ApoE-/-) mice fed high-cholesterol diet	25 mg/kg/day oral TQ for 8 weeks	Down-regulation of LOX-1 expression, reduced serum TC, TGs, and LDL levels	(74)
Clinical Evidence			
Hypertensive patients	2.5 ml of TQ-rich *Nigella sativa* seed oil twice daily orally for 8 weeks	Reduction in serum total cholesterol and LDL levels, and an increase in HDL levels	(76)
Healthy volunteers	200 mg/adult/day oral TQ for 3 months	Significant reduction in serum TC, TG, LDL, and VLDL levels	(77)
Non-alcoholic fatty liver disease patients	1 g oral TQ-rich oil daily for 2 months	Significant reduction in total cholesterol, triglycerides, LDL, and VLDL levels, and an increase in HDL levels	(78)

LDL: low-density lipoprotein, HDL: high-density lipoprotein, VLDL: very low-density lipoprotein, TG: triglycerides, HMGCR: 3-hydroxy-3-methylglutaryl-CoA reductase, mRNA: messenger ribonucleic acid, LDLR: low density lipoprotein receptor, HMG-CoA: β-Hydroxy β-methylglutaryl-CoA, LOX-1: lectin-type oxidized LDL receptor

**Table 3:** Summary of articles reporting on the antihypertensive activity of thymoquinone and *Nigella sativa* seed extracts

Experimental model	Dose/ Duration	Route of administration	Findings	References
*In vivo* Evidence				
L-NAME-induced hypertension in rats	TQ: 0.5 and 1.0 mg/kg BW for 4 weeks	Oral	Dose-dependent decrease in SBP and creatinine Increased glutathione	(87)
Angiotensin II-induced Hypertension	Single dose TQ: 40 mg/kg BW	Intraperitoneal	Decreased SBP, MAP, and HR	(88)
Monocrotaline-induced pulmonary arterial hypertension	TQ: 8 mg/kg for 2 weeks	Oral	Decreased pulmonary arterial pressure and right ventricular hypertension	(89)
High fructose-induced MetS in rats	TQ: 25, 50, and 100 mg/kg for 6 weeks	Oral	Decreased SBP, TBARS Increased SOD, CAT, and GSH	(64)
L-NAME-induced Hypertension in Sprague-Dawley rats	TQ: 2.5, 5, and 10 mg/kg for 4 weeks	Oral	Decreased BP and MAP Increased Aldosterone concentration and ACE activity	(83)
Oxonic acid-induced uricaemia in rats	TQ: 10, and 20 mg/kg BW for 12 weeks		Decreased BP Prevented the accumulation of uric acid, increased mitochondrial ATP	(90)
High fructose-induced MetS in Wistar rats	TQ: 50 mg/kg BW and Sage oil 0.052 m l/kg for 10 weeks	Oral	Decreased BP, BW, BGL, HOMA-IR, TC, TG, and LDL Increased HDL	(17)
L-NAME-induced Hypertension in rats	*N. sativa* oil 2.5 mg/kg BW/ day for 8 weeks	Oral	Prevented increased SBP Decreased cardiac lipid peroxidation, NADPH, ACE activity, and increased Plasma nitric oxide	(84)
DOCA-salt hypertensive and normotensive rats	TQ: 0.25 and 2 mg/kg BW	Intravenous	Decreased BP and HR with a slight decrease in respiratory rate	(91)
Normotensive rats	TQ: 2.5, 5, and 10 mg/kg BW daily for 28 days	Intraperitoneal	A dose-dependent decrease in BP, HR, and MAP	(85)
Clinical Evidence				
Randomized, double-blind, placebo-controlled clinical trial	2.5 m/L *N. sativa* oil for 8 weeks	Oral	Decreased SBP, DBP, TC, TG, and LDL Increased HDL	(76)
Randomized, double-blind, placebo-controlled clinical trial	2.5 m/L *N. sativa* oil twice daily for 8 weeks	Oral	Decreased SBP and DBP with no adverse effects	(92)
Randomized, double-blind, placebo-controlled clinical trial in mild hypertensive patients	100 and 200 mg/kg BW *N. Sativa* seed extract twice daily for 8 weeks	Oral	A dose-dependent decrease in SBP and DBP Decreased TC and LDL	(93)
Randomized, double-blind, placebo-controlled clinical trial with 123 patients	Two crushed/powdered *N. Sativa* seed capsules (500 mg each) twice daily for 6 weeks	Oral	Favorable but not statistically significant decreases in BP due to small sample size	(94)
Randomized, double-blind, placebo-controlled clinical trial pre- and post-test	250 mg twice daily for 6 weeks of *N. sativa *seeds as supplements to simvastatin, metformin, enalapril, atenolol, and clopidgrel	Oral	Decreased BP, FBG, and LDL Increased HDL	(95)
Metabolic syndrome patients open-label study with 90 patients	*N. sativa* seed capsule 500 mg together with amlodipine, atenolol, and atorvastatin for 8 weeks	Oral	Decreased SBP, DBP, and LDL	(86)
Randomized, double-blind, placebo-controlled clinical trial with 20 patients	1000 mg powdered *N. sativa* twice daily for 50 days	Oral	Decreased SBP and DBP Increased HDL	(96)
Single-blind nonrandomized study	2 g of *N. sativa* powder daily for 1 year	Oral	Decreased SBP, DBP, MAP, and HR	(97)
Randomized, double-blind, placebo-controlled clinical trial in elderly patients	300 mg *N. sativa* extract twice daily for 28 days	Oral	Slight decreases in SBP and DBP	(98)

L-NAME: L-Nitro-Arginine Methyl Ester, SBP: Systolic blood pressure, DBP: diastolic blood pressure, MAP: mean arterial pressure, HR: heart rate, BW:body weight, MAPK: mitogen-activated protein kinase, NFκ B: nuclear factor kappa B, PPAR: peroxisome proliferator-activated receptor, α: alpha, γ: gamma, TBARS: thiobarbituric reactive substances, SOD: superoxide dismutase, CAT: catalase, GSH: glutathione peroxidase, BW: body weight, BGL: blood glucose level, FBG: fasting blood glucose, TC: total cholesterol, TG: triglycerides, LDL: low-density lipoprotein, HDL: high-density lipoprotein, ACE: angiotensin-converting enzyme, NADPH: nicotinamide adenine dinucleotide phosphate, ATP: adenine triphosphate

**Table 4:** Role of thymoquinone in insulin signaling, glucose metabolism, and inflammatory adipokines

Study Models	Dosage and treatment	Treatment Outcomes	References
*In vitro* Evidence			
Human THP-1 cell lines	5 and 10 µM of TQ, treatment in cell culture	Reduced risk of atherosclerosis, down-regulation of MCP-1, and ICAM-1 expression	(113)
*In vivo evidence*			
Rat model of high-fructose diet-induced MS	25, 50, and 100 mg/kg of TQ, oral supplementation for 6 weeks	Increased insulin sensitivity and glucose tolerance, up-regulation of PPAR-α and PPAR-γ expression	(64)
Mice with diet-induced obesity (DIO)	20 mg/kg TQ, orally for 24 weeks	Improved insulin signaling, glucose tolerance, and increased pAKT expression via SIRT-1/AMPKα-dependent signaling	(108)
Rats with chronic use of antiretroviral therapy drugs	400 µl/kg TQ, dietary supplementation for 7 months	Prevention of IR associated with antiretroviral therapy drugs	(109)
STZ-induced diabetic rats	50 mg/kg TQ, oral supplementation for 1 month	Up-regulated protein expression and phosphorylation of pAKT in cardiac muscle	(110)
HFD-treated mice	0.75% TQ in combination with 2% ω3 fatty acid, dietary supplementation for 8 weeks	Up-regulated protein expression of adipose tissue browning markers and insulin signaling components	(54)
Rat model of MS fed a Western diet	10 and 20 mg/kg of TQ, oral supplementation for 6 weeks	Significant reduction in HOMA-IR	(111)
Rat model of HFD-induced MS	50 mg/kg TQ and 0.052 ml/kg sage essential oil, oral supplementation for 10 weeks	Reduction in HOMA-IR	(17)
HFD and STZ-induced T2D rats	10 and 20 mg/kg of TQ, daily oral supplementation for 2 weeks	Reduction in HOMA-IR	(68)
Rat model of BPA-induced MS	0.5, 1, and 2 mg/kg of TQ or 21, 42, and 84 μl/kg of TQ-rich *N. sativa* oil, intraperitoneal injection for 54 days	Up-regulation of pIRS, pAKT, and pGSK3 protein expression	(63)
Mice model of DIO	20 mg/kg TQ, oral supplementation for 24 weeks	Reduced serum levels of inflammatory adipokines (resistin and MCP-1)	(108)
HFD-treated mice	0.75% TQ and 2% ω3 fatty acid, dietary supplementation for 8 weeks	Reduced inflammatory adipokine NOV/CCN3 in adipose tissue and liver	(54)
Rat model Olanzapine-induced MetS	2.5, 5, or 10 mg/kg intraperitoneal TQ for 15 days	Amelioration of elevated serum leptin levels	(49)
Rat model of BPA-induced MetS	0.5, 1, and 2 mg/kg of TQ or 21, 42, and 84 μl/kg of TQ-rich *N. sativa* oil, intraperitoneal injection for 54 days	Decreased protein content of leptin, IL-6, and TNF-α in the liver	(113)
Clinical evidence			
Systematic review of clinical studies	Various TQ-rich preparations of *N. sativa* seeds. Daily administration of; Powdered NS seed; 1 g (6 weeks, 12 weeks), 2 g (8 weeks, 12 weeks), 2 g (1 year) NS oil; 1000 mg (8 weeks), 1350 mg (3 months), 3 g (12 weeks), 3 ml (20 days), 5 ml (6 weeks, 2 months, 3 months), oil from 0.7 g seeds (40 days) Water extracts of NS seed; 5 g (6 months) TQ: 50 mg (90 days)	Ameliorative benefits against IR in patients with T2D and related sequelae	(114)

PPAR: peroxisome proliferator-activated receptor, α: alpha, γ: gamma, SIRT 1: sirtuin 1, AMPK: adenosine monophosphate-activated protein kinase, pAkt: phosphorylated Akt, IR: insulin resistance, HOMA-IR: homeostatic model of insulin resistance, pIRS: phosphorylated insulin receptor substrate, GSK3: glycogen synthase kinase-3, MCP-1: monocyte chemoattractant protein-1, NS: Nigella sativa, IL: interleukin, TNF: tumor necrosis factor, ICAM-1: intercellular adhesion molecule-1, T2D: type 2 diabetes

## Conclusion

In conclusion, TQ, a plant-derived phytochemical is the major active ingredient of *N. sativa* which is ingrained in traditional medicinal practices and offers a compelling solution for tackling MetS, a growing global health concern. The abundance of preclinical and clinical data highlighted that the mechanisms of action of TQ are consistent with the multifaceted nature of MetS, making it a potentially valuable alternative and complementary treatment option. However, it is critical to approach the incorporation of TQ into clinical practice cautiously, taking into account factors like dosage, patient selection, and potential drug interactions among others.

The journey from bench to bedside for TQ as an alternative and complementary treatment for MetS is ongoing. Future research endeavors, with a focus on addressing knowledge gaps, exploring combination therapies, and elucidating mechanisms of action, will be pivotal in realizing the full therapeutic potential of TQ. Thymoquinone stands as a beacon of hope, giving a fresh and comprehensive strategy to enhancing the health and well-being of those impacted by this complicated and multifaceted condition, as we work to understand the complexities of MetS and look for viable treatments. For patients struggling with MetS, its incorporation into clinical practice, supported by thorough research and clinical trials, holds the promise of a better and healthier future.
